# Costimulatory molecule‐related lncRNA model as a potential prognostic biomarker in non‐small cell lung cancer

**DOI:** 10.1002/cam4.5391

**Published:** 2022-10-28

**Authors:** Yuanshan Yao, Fuzhi Yang, Anna Chen, Qingwang Hua, Wen Gao

**Affiliations:** ^1^ Department of Thoracic Surgery, Shanghai Key Laboratory of Clinical Geriatric Medicine Huadong Hospital Affiliated to Fudan University Shanghai China; ^2^ Department of Thoracic Oncology Ningbo No. 2 Hospital Ningbo China; ^3^ Ningbo CRRC Times Transducer Technology Co., Ltd. Ningbo China

**Keywords:** costimulatory molecule, non‐small cell lung cancer, prognosis, risk model, LINC01137

## Abstract

**Objective:**

Costimulatory molecules have been demonstrated to exert essential roles in multiple cancers. However, their role in lung cancer remains elusive. Here, we sought to identify costimulatory molecule‐related lncRNAs in non‐small cell lung cancer (NSCLC) and establish a prognostic signature to predict the prognosis of patients with NSCLC.

**Methods:**

A total of 535 lung adenocarcinoma (LUAD) and 502 lung squamous cell carcinoma (LUSC) patients from the cancer genome atlas (TCGA) database were recruited. A novel costimulatory molecule‐based lncRNA prognostic model was constructed using the least absolute shrinkage and selection operator (LASSO) algorithm to predict the overall survival. The Homo_sapiens.GRCh38 data set was set as a reference file for probe annotation.

**Results:**

A total of 593 costimulatory molecule‐related lncRNAs were extracted. After analysis, six costimulatory molecule‐related lncRNAs (AC084859.1, AC079949.2, HSPC324, LINC01150, LINC01150, and AC090617.5) were screened. A prognostic model based on the six lncRNAs was established using systematic bioinformatics analyses. The prognostic model had a prognostic value in NSCLC patients. Furthermore, a prognostic nomogram was established based on clinical parameters and a risk‐score model. Patients with different risk scores had considerably different tumor‐infiltrating immune cells, somatic mutational loading, clinical outcomes, signaling pathways, and immunotherapy efficacy. In addition, LINC01137 was associated with unfavorable disease outcomes and fueled tumor progression in NSCLC.

**Conclusion:**

Taken together, our study demonstrated that a costimulatory molecule‐related lncRNA model could be a potential prognostic biomarker in NSCLC. Moreover, LINC01137 could facilitate the proliferation and invasion of lung cancer.

## Abstract 2

The role of lncRNAs associated with the costimulatory molecular genes in NSCLC is yet to be defined. We hypothesized that costimulatory molecule gene related‐lncRNA might be used as an independent prognosis biomarker, and could lay the basis for a new strategy in the development of novel treatment options for NSCLC.

## 1 INTRODUCTION

Non‐small cell lung cancer (NSCLC), which originates from pulmonary epithelial cells, is a common malignancy worldwide.^1^ NSCLC is mainly divided into two different types: lung adenocarcinoma (LUAD) and lung squamous cell carcinoma (LUSC). Patients with these cancer types bear different pathological characteristics, leading to tumor heterogeneity. As a multifactorial disease, NSCLC has been associated with lifestyle, genetic susceptibility, smoking, etc. Evidence has shown that the incidence of NSCLC is steadily increasing with widespread lifestyle changes.^2^ Surgery remains the best curative option for early‐stage NSCLC. However, early diagnosis is difficult due to the relatively insidious early symptoms of NSCLC. Therefore, most NSCLC patients are at an advanced stage at the time of diagnosis and lose the best chance of surgical resection, resulting in a poor prognosis (Edition 2021). Unfortunately, the therapeutic benefits of advanced‐stage NSCLL are limited. Although chemotherapy is the most commonly used regimen for advanced NSCLC, it has a negligible therapeutic effect.^3^ Currently, immunotherapy using immune checkpoint inhibitors shows promising therapeutic performance and has been the standard treatment for advanced NSCLC.^4^ However, the response rate to immunotherapy is relatively low in most NSCLC patients. Therefore, considering the difference between individual patients, prediction of patient prognosis is difficult.

In recent years, immunotherapy has revolutionized cancer treatment, laying the basis for individualized treatment.^5^, ^6^ Studies have shown that immune checkpoint inhibitors have transformed treatment in patients with advanced NSCLC.^7^ However, only a small proportion of patients have shown satisfactory responses. Approximately 10%–15% of patients showed rapid disease progression, referred to as a hyper‐progressive disease, during treatment with immunotherapy, which might lead to a shorter lifespan. Previous studies have also demonstrated that low expression of important immune signaling molecules and excretion of some cytokines by tumor cells to inhibit the immune response taint the effectiveness of immunotherapy.^8^ Therefore, a better understanding of the mechanism by which CD8T lymphocytes eliminate tumor cells in the tumor microenvironment (TME) is crucial. Killer T cells are activated after the creation of a signal if a major histocompatibility complex on antigen‐presenting cell is distinguished by T cell receptors, followed by dispensing some costimulatory molecules to a second signal.^9^ Costimulatory molecules are crucial in the activation of T cells.^10^ The costimulatory molecules family consists of 61 cell‐surface molecules that regulate the T cell activation and tolerance.^11^, ^12^ The molecules are classified into two groups: the B7‐CD28 family, which consists of well‐known immune checkpoint targets (programmed cell death protein 1 (PD‐1) or its ligand (PD‐L1), CD86/CTLA4) and the TNF family, which includes TNF ligand superfamily and TNF receptor superfamily.^13^, ^14^ Thus, it is theoretically feasible to develop other specific immune checkpoint inhibitors.

Long non‐coding RNAs (lncRNAs) are single‐stranded RNAs that do not encode for any protein.^15^ They are abundant in cells and have been involved in various biological mechanisms, including proliferation, invasion, differentiation, autophagy, and ferroptosis metabolism.^16^, ^17^, ^18^, ^19^, ^20^ Recently, several studies showed that lncRNAs regulate the activity of immune cells, resulting in the alteration of the TME.^21^, ^22^, ^23^ For instance, Qiu et al. found that the ferroptosis‐related lncRNAs could be prognosis markers for colon cancer.^24^ Xie et al. indicated that pyroptosis‐related lncRNAs can effectively predict the prognosis of skin cutaneous melanoma.^25^ Wang et al. demonstrated that signal transducer transcription 3 acts as an lnc‐dendritic cell (DC)‐associated protein that can regulate the maturity of DC and guide DCs to antigens.^26^ Theoretically, lncRNAs can be used as prognostic and diagnostic biomarkers in NSCLC. Useful biomarkers in NSCLC can be easily identified using recently developed high‐throughput sequencing and bioinformatics technologies. A recent study identified a high‐risk model to predict prognosis in LUAD patients based on costimulatory molecule signature genes from the cancer genome atlas (TCGA) and Gene Expression Omnibus profiles.^12^ In addition, a prognosis‐related signature was developed for clear cell renal cell carcinoma, which included 13 costimulatory molecular genes from the TCGA database.^9^ However, these studies only focused on costimulatory molecular genes. The role of lncRNAs associated with costimulatory molecular genes in NSCLC is yet to be defined.

Herein, we utilized RNA sequence data from LUAD and LUSC samples from TCGA to explore costimulatory molecule‐related lncRNAs in NSCLC. Six lncRNAs that are closely associated with prognosis were identified in the TCGA‐LUAD cohort. A costimulatory molecule‐related lncRNA signature was established. Moreover, patients were divided into high‐risk and low‐risk groups based on the median value. Next, differences in the tumor mutation burden (TMB), possible immunotherapy outcomes, and immune infiltration were compared between the two groups. We hypothesized that costimulatory molecule‐related lncRNA may be used as an independent prognostic biomarker. Our findings lay the basis for a new strategy for the development of novel treatment options for NSCLC.

## 2 MATERIALS AND METHODS

### 2.1 Messenger RNA expression data resource

Data were downloaded from the TCGA database, an open‐accessed database that contains whole‐genome sequences spanning 33 cancer types. A total of 535 patients from TCGA‐LUAD and 502 patients from TCGA‐LUSC were recruited. A systemic search of available literature was conducted, and 61 costimulatory molecule genes were identified.^12^ The lncRNA profile was extracted based on the biotype of Homo_sapiens.GRCh38 file extracted from ENSEMBL (http://asia.ensembl.org/index.html).^27^ The Limma package was used to distinguish lncRNAs and protein‐coding genes.^28^ LncRNAs with a correlation coefficient >0.4 and *p* < 0.01 were defined as costimulatory molecule‐related lncRNAs. GSE138172 with an lncRNA expression profile of five paired lung cancer and adjacent normal tissue was used to validate model lncRNAs. GPL16956, the platform of GSE138172, was re‐annotated by sequence alignment.

### 2.2 Establishment of a prognostic model for NSCLC

Univariate Cox regression analysis was performed to identify prognosis‐related lncRNAs using a *p* < 0.01 threshold. The least absolute shrinkage and selection operator (LASSO) and multivariate Cox regression analyses were performed to screen for potential prognosis‐related lncRNAs. The risk score was then calculated using the following formula: Risk score = β1 * Exp1 + βi * Expi. β represents the coefficients while Exp represents the extent of expression of the genes.^29^ The TCGA‐LUAD cohort was the training group and TCGA‐LUSC was the validation group. Based on the median cutoff point, LUAD patients (training group) were divided into high‐risk or low‐risk groups. The prediction efficiency of the prognostic model was verified using LUSC patients (validation group). The six prognostic lncRNAs were used to perform the Kaplan–Meier analysis. Clinical indexes were gathered, and R software was used to construct a prognostic nomogram to predict NSCLC. Then, prediction accuracy of this nomogram for 1‐, 3‐, and 5‐year overall survival (OS) was validated.

### 2.3 Immune infiltration

Single‐sample gene set enrichment analysis (ssGSEA) was used to assess the immune infiltration abundance in NSCLC and analyze the landscape of 24 different types of immune cells.^30^ Results were visualized in a bubble chart. The correlation between the risk score model and the expression level of immune checkpoint‐related genes was further analyzed in the high‐risk and low‐risk groups.

### 2.4 Mutation analysis

Mutation profile analysis was performed in low‐risk and high‐risk groups of the TCGA‐LUAD cohort. TMB and possible responses to PD‐1 and CTLA‐4 immune checkpoint inhibitors were compared between the two groups.

### 2.5 Biological signaling enrichment analysis

GSEA was performed to detect biological processes perturbed in the two groups using a hallmark gene set.^31^

### 2.6 Patient specimens and cell culture

Cell lines BEAS‐2B, A549, H1299, H23, and H520 were obtained from Shanghai ExCell Biology, Inc. A total of 123 non‐small lung cancer tissues and 8 randomly selected corresponding normal tissues were obtained from patients at Ningbo No. 2 Hospital from November 2014 to December 2021. A total of 77 patients were males and 46 were females, with a mean age of 52.0 ± 9.7 years. This study was approved by the Ethics Committee of Ningbo No. 2 Hospital and written informed consent was obtained from each subject. The detailed clinical information of the 123 patients was shown in Appendix S1.

### 2.7 Quantitative real‐time polymerase chain reaction (qRT‐PCR)

Total RNA from cancer tissues, normal tissues and transfected cells was extracted using TRIzol (GIBCO). After reverse transcription, qRT‐PCR was performed on an ABI 7500 Real‐Time PCR System (Applied Biosystems). The 2 − ΔΔCt method was used to assess the expression levels of the RNAs. The primers used were as follows: LINC01137, forward primer: 5′‐CGCAAGATAAGCACGGACTG‐3′, reverse primer: 5′‐GCTTCATCAGGCAGGGTGTA‐3′, GAPDH, forward primer: 5′‐GGAGCGAGATCCCTCCAAAAT‐3′, reverse primer: 5′‐GGCTGTTGTCATACTTCTCATGG‐3′, HSPC324, forward primer: 5′‐CTGGAGCCTCAGAGGCAGAGC‐3′, reverse primer: 5′‐GCACTCACACGCCATCTGTGG‐3′, AC090617.5, forward primer, 5′‐TAATGTAAGCATCGGGGTCTTG‐3′, reverse primer, 5′‐GCATGGTGATGCATGACTGTC‐3′, LINC00150, forward primer, 5′‐GATGGAGTCGCTCTGGTTCAC‐3′, reverse primer, 5′‐ATACCTATCCCGCAATGTTGT‐3′, AC084859.1, forward primer, 5′‐AACAAGATTCGGCAATGTGCT‐3′, reverse primer, 5′‐CGCCACTGTGAAAACTCCTATC‐3′, AC079949.2, forward primer, 5′‐TTGCTGCTTATGACTGCTGAG‐3′, reverse primer, 5′‐GGTATCTGAATCCAAATTGTGCT‐3′.

### 2.8 Cell transfection

Negative control (NC) LINC01137 and short hairpin RNAs (shRNAs) specifically targeting LINC01137 were purchased from RiboBio. A549 and H1299 cells were transfected with NC LINC01137 or shRNA‐LINC01137 using Lipofectamine 3000 (Invitrogen).

### 2.9 Colony formation and EdU assay

A549 and H1299 cells were simultaneously transfected and seeded into 6‐well plates with 10% phosphate‐buffered saline. After 14 days, colony cells were stained with 1% crystal violet for 20–30 min, and then counted. After transfection of NC LINC01137 and shRNA‐LINC01137 into the A549 and H1299 cells, respectively, the EdU assay and 4′,6‐diamidino‐2‐phenylindole (DAPI; RiboBio) were used to investigate cell proliferation, following the manufacture's protocols. The nuclei double‐stained with EdU and DAPI were calculated.

### 2.10 Transwell migration and invasion assays

Transwell invasive and migratory capacity of A549 and H1299 cells. Cells were inserted into 24‐well transwell plates (Corning) without Matrigel for migration assay and with Matrigel (Corning) for invasion assay, respectively. After 48 h, cells that had invaded the lower chamber were stained with 0.1% crystal violet for 30 min and counted.

### 2.11 Statistical analysis

qRT‐PCR data were compared using one‐way ANOVA. Statistical analyses were calculated in R and Bioconductor packages (version 4.0.5). *p* < 0.05 was considered statistically significant.

## 3 RESULTS

### 3.1 Identification of costimulatory molecule‐related lncRNAs

**FIGURE 1:**
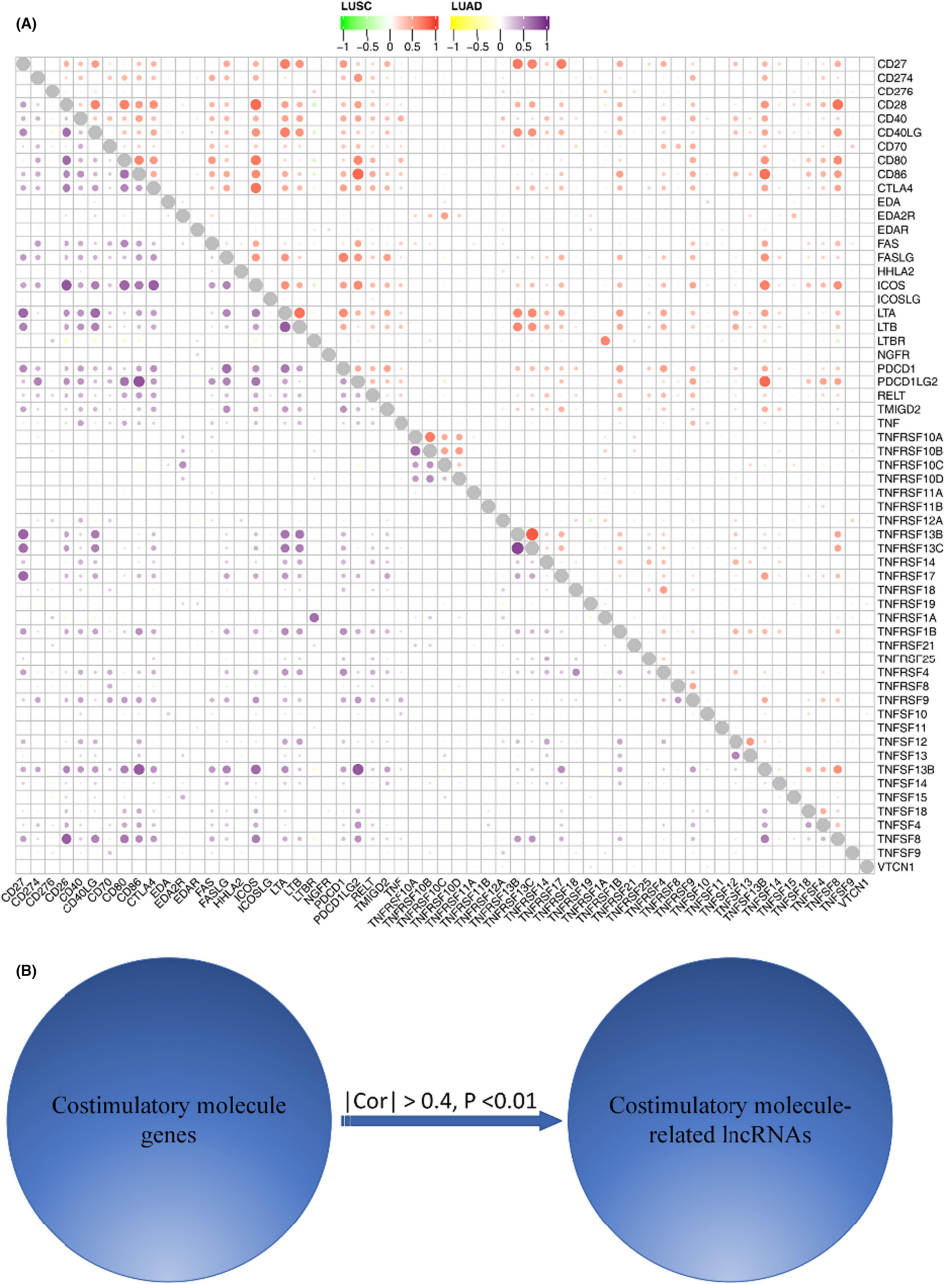
Confirmation of costimulatory molecule‐related lncRNAs in LUAD patients. (A) Co‐expression of costimulatory molecule genes between TCGA‐LUAD and TCGA‐LUSC. (B) Pearson correlation analysis of 593 costimulatory molecule‐related lncRNAs.

The flow chart of the whole study was shown in Figure S1. Data can be downloaded from the https://figshare.com/articles/dataset/data/21085519 website. A total of 61 costimulatory molecule genes co‐expressed in both TCGA‐LUAD and TCGA‐LUSC were identified (Figure 1A). In addition, 593 costimulatory molecule‐related lncRNAs were identified using Pearson correlation analysis (|R| > 0.4, *p* < 0.01, Figure 1B).

### 3.2 Establishment and validation of a costimulatory molecule‐related lncRNA model

**FIGURE 2:**
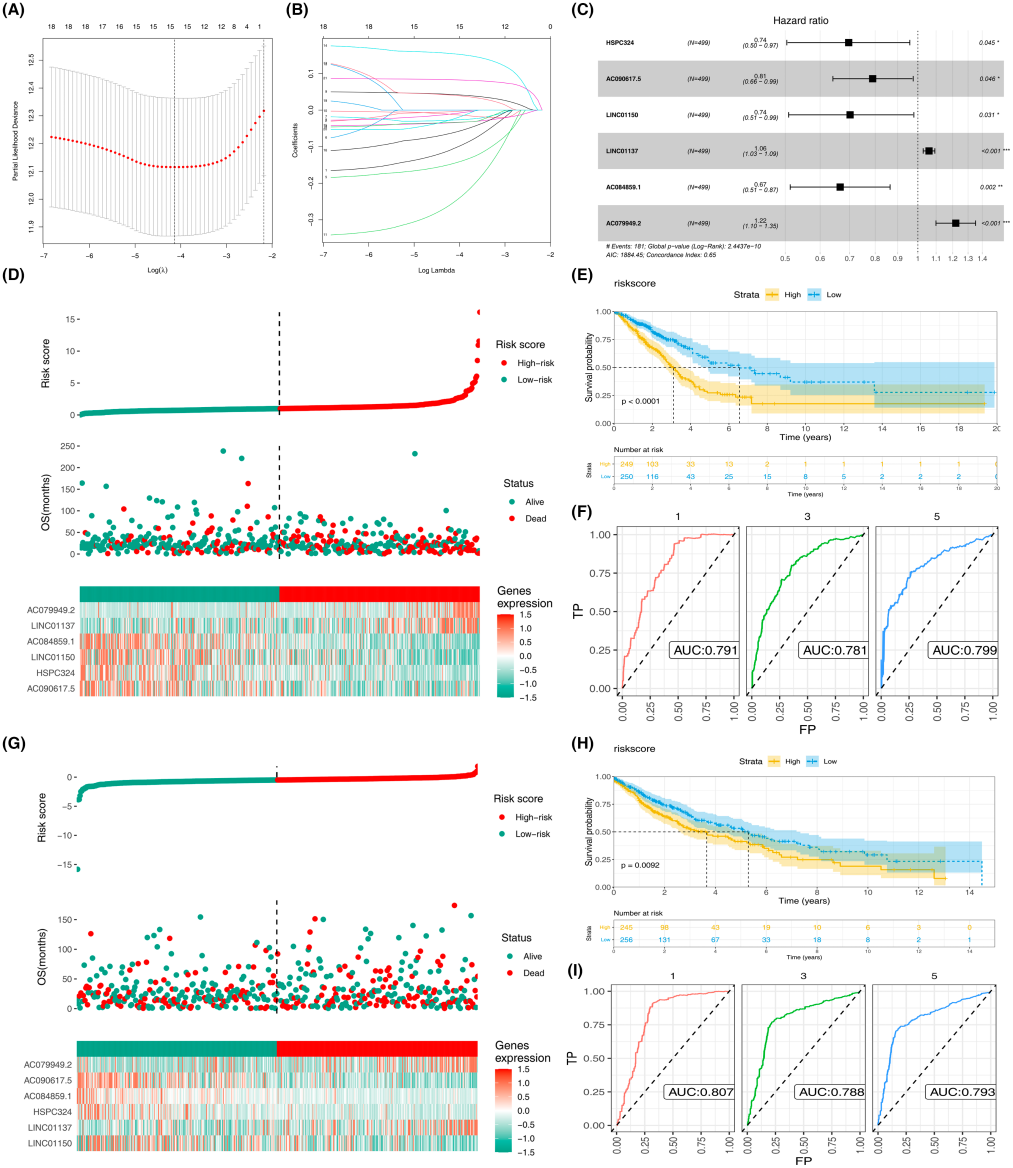
Construction of a six lncRNA prognostic model in NSCLC patients. (A, B) Six prognostic lncRNAs were extracted. (C) Establishment of the risk score model. (D–F) Validation of the risk score model in LUAD patients. (G–I) Verification of the risk in LUSC patients.

Univariate COX regression analysis was performed to determine the potential prognostic value of the 593 lncRNAs. Only 21 prognosis‐related lncRNAs were calculated (Table 1). LASSO regression analysis was then performed for dimensionality reduction (*p* < 0.01, Figure 2A,B). Six prognostic lncRNAs, including HSPC324, AC090617.5, LINC01150, LINC01137, AC084859.1, and AC079949.2, were identified using multivariate Cox analysis. A costimulatory molecule‐related lncRNA model was established and the risk score was defined as HSPC324 * −0.304 + AC090617.5 * −0.211 + LINC01150 * −0.296 + LINC01137 * 0.058 + AC084859.1 * −0.405 + AC079949.2 * 0.198 (Figure 2C).

**TABLE 1:** Univariate cox regression analysis of prognosis‐related lncRNAs

ID	HR	HR.95 L	HR.95H	*p*‐value
LINC00941	1.120	1.076	1.166	≤0.001
AC079949.2	1.286	1.168	1.415	≤0.001
AP000695.2	1.417	1.193	1.683	≤0.001
HLA‐DQB1‐AS1	0.926	0.889	0.963	≤0.001
AP000695.1	1.256	1.113	1.417	≤0.001
LINC01137	1.058	1.026	1.092	≤0.001
AC090559.1	0.790	0.682	0.914	≤0.001
AC011477.2	0.830	0.736	0.935	≤0.001
AC005332.4	0.728	0.594	0.893	0.002
LINC01150	0.577	0.405	0.823	0.002
AC084859.1	0.666	0.510	0.870	0.003
AL034397.3	0.751	0.621	0.909	0.003
LINC00996	0.570	0.392	0.829	0.003
HSPC324	0.585	0.406	0.841	0.004
AC024075.1	0.801	0.687	0.933	0.004
AC090617.5	0.744	0.607	0.912	0.004
AC024075.3	0.729	0.582	0.913	0.006
AC004687.1	0.865	0.779	0.960	0.006
AF131215.5	0.725	0.572	0.919	0.008
PCBP1‐AS1	0.499	0.298	0.836	0.008
CARD8‐AS1	0.829	0.719	0.956	0.009

The TCGA‐LUAD cohort was used as the training set; LUAD patients were then classified into high‐risk and low‐risk groups based on the median risk score (Figure 2D). Kaplan–Meier analysis showed a significant difference between the two groups (*p* < 0.0001, Figure 2E). In TCGA‐LUAD cohort, 1‐, 3‐, and 5‐year area under curves (AUCs) were 0.791, 0.781, 0.799, respectively (Figure 2F). The value of the risk model was then verified in the LUSC cohort. LUSC patients were categorized into high‐risk and low‐risk groups based on the same prognostic risk score (Figure 2G). The Kaplan–Meier curve showed that the high‐risk group had a worse prognosis compared with the low‐risk group (Figure 2H). The receiver operating characteristic AUCs for 1‐, 3‐, and 5‐year survival rates were 0.807, 0.788, and 0.793, respectively (Figure 2I). These results showed that our risk model possessed good prognostic performance for both the training and validation samples.

Further, the performance of our model was compared with other previously published lncRNA biomarkers in NSCLC.^32^, ^33^, ^34^, ^35^ The results showed that in the LUAD cohort, our model showed better performance than the signature identified by Zhou et al., Yang et al., and Ren et al. (Our model, AUC = 0.791; Zhou et al., AUC = 0.713; Yang et al., AUC = 0.619; Ren et al., AUC = 0.696; Figure S2A). However, in the NSCLC cohort, our model was inferior to the lncRNA signature identified by Zhou et al. (Our model, AUC = 0.703; Zhou et al., AUC = 0.720; Figure S2B). Moreover, univariate and multivariate Cox regression analyses of the model were performed in the whole NSCLC cohort and the result showed that our model was an independent risk factor for other clinical features (Figure S3A,B).

### 3.3 Relationship between the prognostic model and clinicopathological features of NSCLC

**FIGURE 3:**
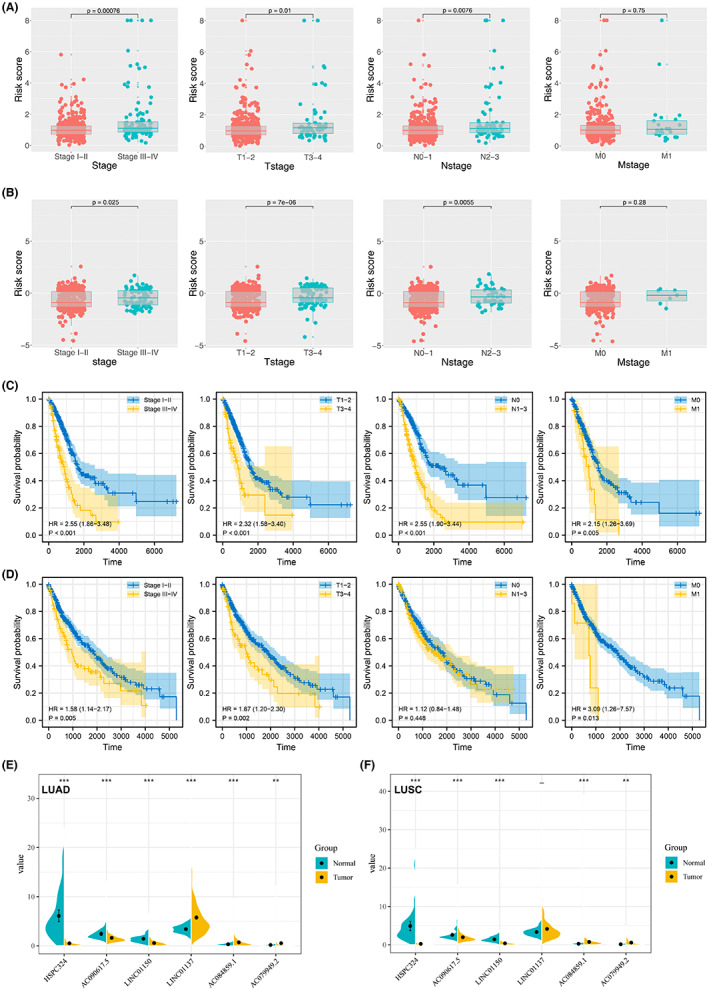
Association between TNM and the risk score coupled with the expression of the six lncRNAs in normal and NSCLC tissues. (A, B) Association between TNM and the risk score in LUAD and LUSC patients, respectively. (C) KM survival curves of patients with different clinical features in LUAD patients. (D) KM survival curves of patients with different clinical features in LUSC patients. (E, F) Expression of the six prognostic lncRNAs in normal and cancer tissues in LUAD and LUSC cohorts. **p < 0.01, and ***p < 0.001.

Clinical stage, T stage and N stage were significantly associated with a low‐risk score (*p* < 0.05) (Figure 3A). However, no significant difference in the M stage was observed between the high‐risk and low‐risk groups. The same trend was also observed in the LUSC cohort (Figure 3B). KM survival curves showed that in LUAD patients, the patient with more progressive clinical features might have a worse prognosis (Figure 3C, Stage III‐IV vs. Stage I‐II, T3‐4 vs. T1‐2, N1‐3 vs. N0, M1 vs. M0). The same conclusion was also observed in the LUSC patients (Figure 3D). LINC01137, AC084859.1, and AC079949.2 were overexpressed whereas HSPC324, AC090617.5, and LINC01150 were significantly downregulated in cancer tissues compared with normal tissues of LUAD patients (*p* < 0.05, Figure 3E). The six prognostic lncRNAs were then validated in LUSC samples. The relative expression between the normal and tumor tissues in five prognostic lncRNAs (AC084859.1, AC079949.2, HSPC324, LINC01150, LINC01150, and AC090617.) of LUSC samples showed a similar trend to those in the LUAD cohort (*p* < 0.05. Figure 3F). Meanwhile, the result of GSE138172 indicated that HSPC324 was downregulated, while AC084859.1 was upregulated in NSCLC tissue. These results were consistent with those of TCGA (Figure S4). However, no significant difference was observed in AC079949.2, AC090617.5, and LINC00150 (Figure S4). Based on our tissues, we found that AC084859.1 and LINC00150 was upregulated, while HSPC324 was downregulated in lung cancer tissue (Figure S5). However, no significant difference was observed in AC079949.2 and AC090617.5 (Figure S5).

### 3.4 Kaplan–Meier curves based on the TCAG‐LUAD cohort

**FIGURE 4:**
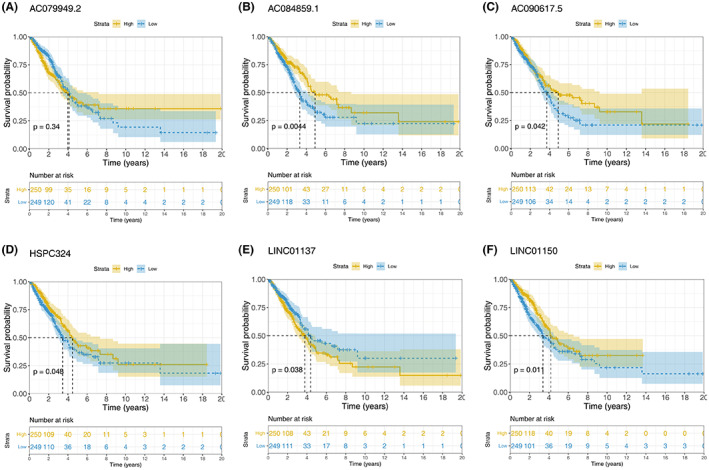
Kaplan–Meier curves with log‐rank tests based on these six prognostic lncRNAs in the TCGA‐LUAD patients.

Kaplan–Meier curves with log‐rank tests were drawn based on the six prognostic lncRNAs in the TCGA‐LUAD cohort (Figure 4A–F). As is shown in Figure 4A–F, higher expression of AC084859.1, AC090617.5, HSPC324, and LINC01150 was correlated with superior OS. Besides, higher expression of LINC01137 was associated with inferior disease prognosis. Moreover, the expression profile of AC079949.2 had no significant effect on the OS.

### 3.5 Construction of a prognostic nomogram model based on the risk score and Clinicopathologic factors

**FIGURE 5:**
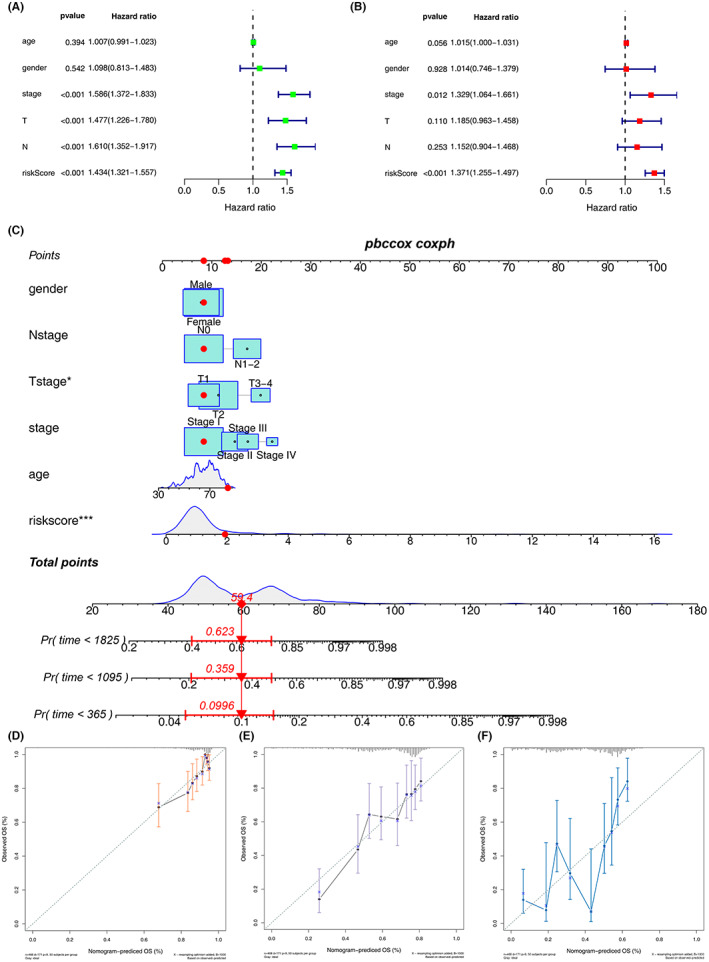
Prognostic nomogram based on the risk score coupled with clinicopathologic features. (A, B) Risk score was an independent risk factor in LUAD and LUSC patients. (C) Establishment of a prognostic nomogram based on the risk score combined with clinicopathologic features of LUAD patients. (D) Calibration curves for 1‐, 3‐ and 5‐year overall survival showed good consistency between the nomogram predictions and the actual observed outcomes in the LUAD samples, respectively.

Univariate and multivariate Cox regression analyses were conducted to explore the risk score and some clinicopathologic indexes. Collectively, the risk score, T stage, stage, and N stage (all *p* < 0.001) were negatively associated with the OS in LUAD patients (Figure 5A). Similarly, the risk score (*p* < 0.001) and stage (*p* < 0.05) yielded unfavorable outcomes in LUSC samples (Figure 5B). Based on the above factors, a nomogram model was successfully established (Figure 5C). The calibration curves for 1‐, 3‐, and 5‐year OS showed good consistency between the nomogram predictions and the actual observed outcomes in the LUAD samples (Figure 5D‐F).

### 3.6 Evaluation of tumor immune microenvironment

**FIGURE 6:**
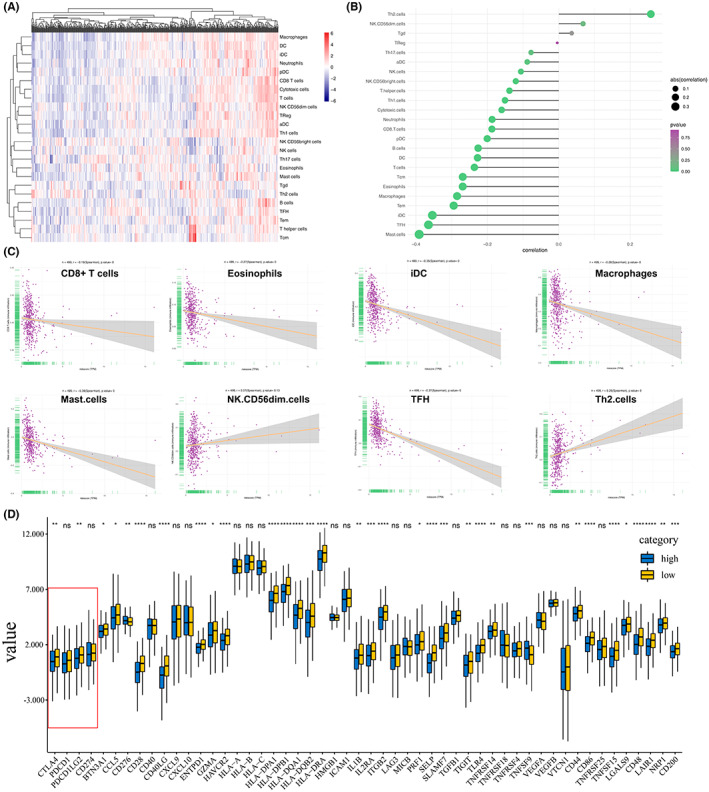
Immune landscape and expression level of immune checkpoint‐related genes between the high and low score groups. (A–C) Immune cell infiltration in the high‐score LUAD patients. (D) Expression levels of immune checkpoint‐related genes between the two groups. *p < 0.05, **p < 0.01, ***p < 0.001.

The immune cell landscape was calculated using the ssGSEA algorithm (Figure 6A). The results revealed that among the immune cells, T helper type 2 (Th2) cells displayed relatively higher expression in high‐risk patients (*p* < 0.001). Conversely, significant immune cells such as CD8 + T cells, eosinophils, immature DC (iDC), Mast and T follicular helper (TFH) were negatively associated with the risk score (*p* < 0.001, Figure 6B,C). Moreover, the expression levels of immune checkpoint‐related genes between the two groups were investigated. As shown in Figure 6D, the expression levels of the top four genes, including PD‐1 (PDCD1), CTLA4, PD‐L1 (CD274), and PD‐L2 (PDCD1LG2), were significant because they have been shown clinically to correlate with immunotherapeutic responsiveness.^36^ CTLA‐4 and PD‐L2 were highly expressed in the low‐risk group (*p* < 0.01).

### 3.7 Somatic mutations in the two groups

**FIGURE 7:**
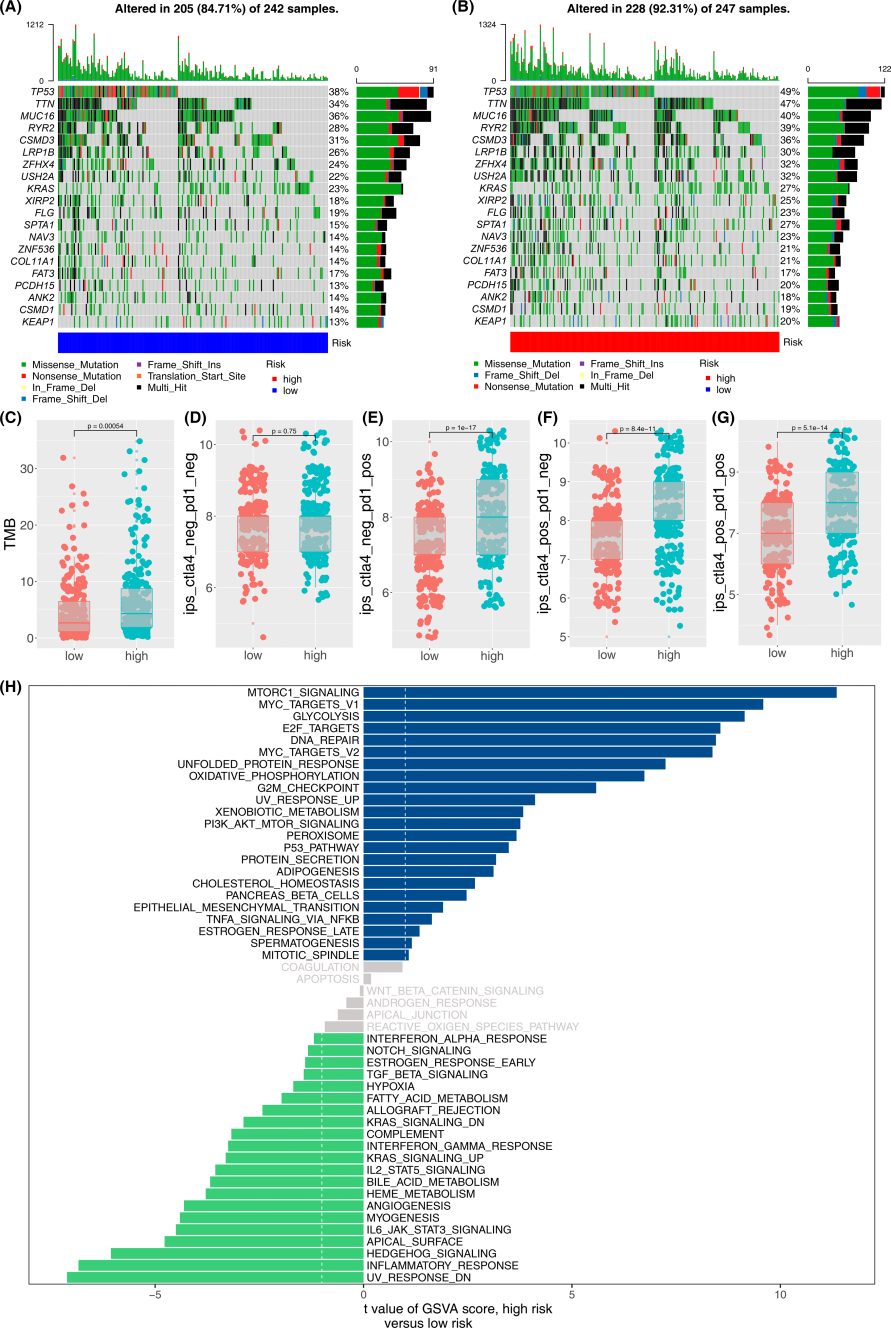
Somatic mutation, possible response to immunotherapy and enriched pathways between the high‐risk and low‐risk groups. A‐B. Heatmap of somatic mutation in the two groups. C‐G. Possible response to immunotherapy between the high‐risk and low‐risk groups. H. Enriched pathways in the two groups.

The distribution of somatic mutations was profiled in the two groups (Figure 7A,). The high‐risk group had high‐gene mutation (>25%) of TP53 (49%), TTN (47%), MUC16 (40%), RYR2 (39%), CSMD (36%), LRP1B (30%), ZFHX4 (32%), USH2A (32%), KRAS (27%), and XIRP2 (25%). The low‐risk group was characterized by frequent mutation (>25%) of TP53 (38%), TTN (34%), MUC16 (36%), RYR2 (28%), CSMD (31%), and LRP1B (26%). In addition, TMB was higher in the high‐risk group (*p* < 0.001, Figure 7C), which may explain the shortened OS in the high‐risk cohort.^37^

Furthermore, the potential response to immunotherapy between the two groups was assessed. As shown in Figure 7D, no significant difference was observed in patients without immunotherapy between the high‐risk and the low‐risk group. Our data showed that patients in the high‐risk group may respond better to PD‐1 immunotherapy than those in the low‐risk group (Figure 7E). Similarly, the result showed that patients in the high‐risk group may benefit from CTLA‐4 immunotherapy than those in the low‐risk group (Figure 7F). Collectively, high‐risk group patients are most likely to benefit from embracing CTLA‐4 and PD‐1 dual immunotherapy (Figure 7G).

### 3.8 Enrichment of biological pathways in the two risk groups

To explore the potentially enriched biological pathways in LUAD, the most enriched terms of biological pathways were selected using GSEA. The data showed that “mTORC1,” “MYC,” “GLYCOLYSIS,” “E2F,” and “DNA‐REPAIR” were enriched in LUAD samples with high‐risk scores. “UV‐RESPONSE‐DN,” “INFLAMMATORY,” “HEDGEHOG,” “APICAL,” and “IL‐6JAK‐STAT3” were enriched in the low‐risk group (Figure 7H).

### 3.9 LINC01137 promotes NSCLC proliferation and invasion

**FIGURE 8:**
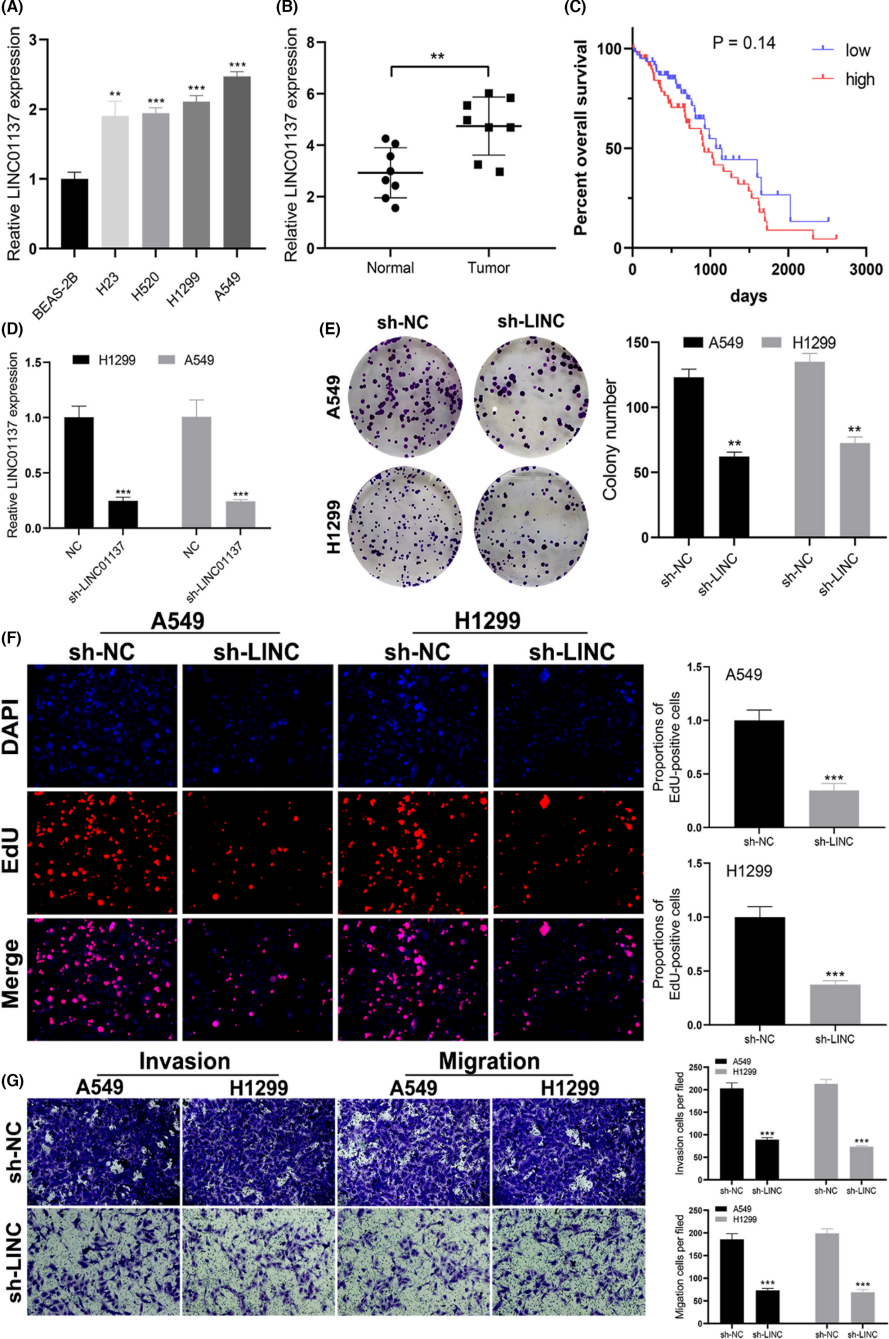
LINC01137 is a tumor‐promoting gene in NSCLC. (A) LINC01137 expression profile in the normal lung cell and NSCLC cell lines. (B) LINC01137 expression in normal and tumor tissues. (C) Kaplan–Meier curve based on the expression of LINC01137 in NSCLC patients. (D) qRT‐PCR analysis of transfection efficacy of LINC01137. (E–G) Effect of LINC01137 knockdown on the proliferation, invasion, and migration of A549 and H1299 cells. **p < 0.01, ***p < 0.001.

LINC01137 expression was significantly upregulated in NSCLC cell lines A549, H1299, H23, and H520 compared with human normal lung cells BEAS‐2B (Figure 8A). In 8 paired tumor and normal tissues, LINC01137 was overexpressed in the tumor tissues compared with healthy tissues (*p* < 0.05, Figure 8B). Although the Kaplan–Meier analysis showed that there was no statistically significant difference in the OS between the high and low LINC01137 expression, patients with low expression of LINC01137 were likely to exhibit better outcomes (Figure 8C). qRT‐PCR revealed that the LINC01137 expression level in NSCLC cells transfected with shRNA‐LINC01137 was significantly lower than that in the NC group (*p* < 0.05; Figure 8D). Colony formation assay data indicated that the number of colonies was significantly reduced after transfection with sh‐LINC01137 in NSCLC cells (Figure 8E). EdU assay also revealed that proliferation of NSCLC cells was notably impaired due to LINC01137 downregulation (Figure 8F). Further, knockdown of LINC01137 inhibited the invasion and migration of NSCLC cells (Figure 8G).

## 4 DISCUSSION

The incidence rate of lung cancer is on the rise worldwide and has attracted considerable attention. Although immunotherapy has revolutionized treatment in advanced‐stage NSCLC patients and become the mainstay treatment option for eligible patients, its prognosis remains poor.^38^, ^39^ However, only a small proportion of advanced‐stage NSCLC patients have been shown to benefit from immunotherapy.^40^ Unfortunately, about 8%–15% of treated patients experienced unexpected hyper‐progressive disease after injection with PD‐1/PD‐L1 inhibitors, which worsened clinical outcomes.^41^ The variability of responses to immunotherapy emphasizes the need to define the possible underlying causes. The complexity of the TME demands that important factors such as cytokine, chemokine, and costimulatory molecules^8^, ^42^ must be considered to obtain a better response with PD‐1/PD‐L1 inhibitors. PD‐1/PD‐L1 and CTLA‐4 are negative costimulatory molecules; some positive costimulatory pathways are also crucial in the activation, proliferation, and recruitment of CD8 T cells.^10^, ^43^ However, molecular functions and biological pathways of these costimulatory molecules in NSCLC are yet to be explored. The functions of lncRNA in immune regulation have also attracted huge research interest.^44^ Thus, a better understanding of the immune landscape would help improve outcomes in NSCLC patients. Dual immunotherapy could activate and motivate CD8T and memory T cells more persistently.^45^ To optimize immunotherapy and obtain valuable clues for the prediction of NSCLC prognosis, there is a need to explore some costimulatory molecules, which may guide immunotherapy and the discovery of novel biomarkers for NSCLC.

In the present study, we simultaneously extracted 61 costimulatory molecules and multiple lncRNAs from the TCGA database.^12^, ^46^ After Pearson correlation analysis, 594 costimulatory molecule‐related lncRNAs were obtained. Ultimately, six novel prognostic lncRNAs associated with the outcome of the TCGA‐LUAD cohort were extracted, and then the risk score was estimated. This risk score was then verified in the TCGA‐LUSC cohort. Interestingly, this signature could be used as a universal model for both LUAD and LUSC patients. It has been shown that LUAD and LUSC share distinct biological features and tumoral heterogeneity.^47^ We demonstrated that the risk score may serve as a new biomarker of both LUSC and LUAD.^48^ Moreover, the reliability of our risk score was strengthened by its high correlation with the T stage, N stage, and TNM stage in both LUSC and LUAD patients. Out of the 6 prognostic lncRNAs in the LUAD cohort, LINC01137 significantly differed between cancer and healthy tissues. In addition, LINC01137 was an independent risk factor in the TCGA‐LUAD cohort (*p* = 0.038, Figure 4E). Therefore, LINC01137 was used to explore tumor‐promoting roles in LUAD patients and cells. To increase the robustness of the prognostic signature, a new prognostic nomogram was established based on both clinicopathological indexes and the risk score, which may not only assist in forecasting the OS of lung cancer patients but also supply patients with individualized treatments.

The TME consists of tumor cells, multiple immune cells, tumor stroma and various cytokines and chemokines.^49^ Tumor‐infiltrating immune landscapes are closely associated with the OS of patients.^50^ Thus, both tumor cells and stroma can secrete intercellular factors to regulate the immune cell landscape and modulate tumor persistence.^51^ Our data showed that there were lower percentages of CD8T, eosinophils, iDC, mast, macrophages and TFH cells and a higher percentage of Th2 cells in the high‐score patients (*p* < 0.05). Furthermore, altered Th1/Th2 cell ratios reflect apparent immune evasion and have been considered an advanced stage of tumor progression and invasion.^52^ This suggests that our risk score signature may play a role in the immune cell infiltration landscape.^11^, ^53^ The differences in the expression level of costimulatory molecule genes, including PD‐L1, CTLA4, PD‐1, and PD‐L2, were also compared between the two groups. PD‐1 can bind two cell‐surface proteins: PD‐L1 and PD‐L2. It has been proved that both proteins compete for binding to PD‐1. PD‐L2 binds to PD‐1 with a three‐fold stronger affinity compared with PD‐L1.^54^ Previous data showed that PD‐L1 expression is more prevalent than PD‐L2 in cancer cells.^55^ This may explain why some patients with PD‐L1 stain negative but may benefit from PD‐1 inhibitor. The expression level of CTLA4 and PD‐L2 genes was lower in the high‐risk group, implying that the two groups might differentially respond to immunotherapy.

High‐risk patients seem to exhibit a higher TMB than low‐risk patients. A higher TMB indicates that more neo‐antigens are transferred to T cells, and thus, better immunotherapy efficacy.^40^ Meanwhile, TP53 and TTN had the highest mutation frequency in both groups. Interestingly, Jia et al. showed that TP53 coupled with TTN mutation is another potential biomarker for predicting immunotherapy outcomes.^56^, ^57^ Next, the potential immune response was determined between the two groups. However, it is unreliable to forecast the possibility of immunotherapy based on one factor and thus there is a need to consider several factors, including host‐intrinsic factors, tumor‐intrinsic factors, and TME.^58^ Collectively, high‐risk patients exhibited a better response to CTLA‐4 and PD‐1 checkpoint inhibitors compared with low‐risk patients. These results robustly demonstrated that high‐risk patients possess a relatively immune‐inflamed microenvironment and high mutational burden, resulting in a relatively better response to immunotherapy.^59^ However, clinical trials are needed to confirm these findings.

In the Kyoto Encyclopedia of Genes and Genomes (KEGG) analysis, these prognostic lncRNAs were mainly enriched in biological pathways, including mTORC1, MYC, and GLYCOLYSIS. These pathways are often involved in the regulation of expression levels of a costimulatory molecule. For instance, MYC signaling participates in the regulation of PD‐1 in NSCLC.^60^, ^61^

Furthermore, in vitro assays showed that LINC01137, a prognostic lncRNA, promoted tumor progression. The results of qRT‐PCR showed that the expression of LINC01137 was enhanced in NSCLC tissues and NSCLC cell lines. In addition, we showed that LINC01137 knockdown decreased the proliferation, migration, and invasion of NSCLC cells in colony formation, EdU assay and transwell assays. Thus, LINC01137 plays an oncogenic role in NSCLC, and its overexpression accelerates tumor growth. These findings are consistent with a recent study that demonstrated that LINC01137 acts as a tumor promoter in oral squamous cell carcinoma.^62^ Although our data did not achieve a significant difference in OS between high and low LINC01137 expression groups, it was found that patients with low expression of LINC01137 were likely to exhibit better outcomes. Thus, a large‐scale study is needed to confirm the LINC01137 expression level associated with prognosis in NSCLC patients.

Recent studies showed that LINC01137 modulates several other metabolic pathways such as redox, autophagy, oxidative stress, and ferroptosis.^63^, ^64^, ^65^, ^66^ Thus, the biological functions of LINC01137 warrant further exploration. A better understanding of the biology of lncRNA could improve our knowledge of multiple cell functions and diseases, and present new avenues for the development of unique and novel therapeutic strategies.

However, this study has several limitations. As a retrospective design, this study is likely to be biased. Besides, based on different biomarkers used to assess the efficacy of immunotherapy, the response of the high‐risk group to immunotherapy may be evaluated indirectly and the rate of accuracy might fluctuate. Therefore, these data could be validated by analyzing clinical samples.

## 5 CONCLUSION

In summary, the present study conducted a comprehensive analysis of costimulatory molecule‐related lncRNAs in NSCLC patients and extracted six lncRNAs with a prognostic value. A novel prognostic model for NSCLC patients was then successfully established and validated using the six lncRNAs. This prognostic model stratified patients into two groups with different prognoses and correlated with clinical features, tumor‐infiltrating immune cells, somatic mutational loading, clinical outcomes, signaling pathways, and immunotherapy efficacy. Moreover, our findings demonstrated that LINC01137 could facilitate the proliferation and invasion of lung cancer.

## AUTHOR CONTRIBUTIONS

**Yuanshan Yao:** Conceptualization (supporting); writing – original draft (lead); writing – review and editing (supporting). **Fuzhi Yang:** Validation (equal); writing – review and editing (equal). **Anna Chen:** Software (lead); supervision (lead). **Qingwang hua:** Data curation (equal); methodology (lead); resources (lead). **Wen Gao:** Formal analysis (lead); investigation (lead); project administration (lead); supervision (lead).

## FUNDING INFORMATION

This study was supported by the Shanghai Key Laboratory of Clinical Geriatric Medicine, Shanghai Municipal Key Clinical Specialty (shslczdzk02801), Digital Technologies for the postoperative remote care and rehabilitation of thoracic and cardiac surgery patients (2019YFE0105600) and Zhejiang Provincial Natural Science Foundation of China under Grant No. LQ22H160059.

## CONFLICTS OF INTEREST

The authors declare that they have no competing interests.

## ETHICS APPROVAL AND CONSENT TO PARTICIPATE

Ethics Statement: The study was conducted following the Declaration of Helsinki (as revised in 2013). The study was approved by the Ethics Committee of Ningbo No. 2 Hospital, and individual consent for this retrospective analysis was obtained.

## CONSENT

Written informed consent was obtained from each patient. A copy of the consent is available with the editorial office.

## Supporting information


Figure S1
Figure S2
Figure S3
Figure S4
Figure S5

Appendix S1

## Data Availability

All data analyzed during this study are included in this published article.
