# Supplementary material for: Costimulatory molecule‐related lncRNA model as a potential prognostic biomarker in non‐small cell lung cancer
Source: Cancer Med. 2022 Oct 28;12(5):6419–36. doi: 10.1002/cam4.5391 (PMC10028169; doi:10.1002/cam4.5391)
Supplement: Supplementary file 1 — Figure S1 Figure S2 Figure S3 Figure S4 Figure S5 [file CAM4-12-6419-s001.docx]

Figure S1. The flow chart of the whole study.

Figure S2. Performance of our model with other previously published lncRNAs biomarkers in NSCLC. A. Our model showed better performance than the signature identified by other previously published lncRNAs biomarkers in the LUAD cohort. B. Our model was inferior to the lncRNAs biomarker explored by Zhou et al in the NSCLC cohort.

Figure S3. Assessment of the value of the risk score of the 6 lncRNAs. A. Univariate Cox regression analysis of the association between the overall survival and clinicopathological features. B Multivariate Cox regression analysis showed that the risk signature was an independent predictive factor for the overall survival of patients.

Figure S4. Expression of other five prognostic lncRNAs in normal and cancer tissues in LUAD and LUSC cohorts.

Figure S5. Expression of other five prognostic lncRNAs in normal and cancer tissues based on eight paired tumor and healthy tissues
